# Effect of obesity, lipids and adipokines on allergic rhinitis risk: a Mendelian randomization study

**DOI:** 10.1016/j.bjorl.2023.101306

**Published:** 2023-08-17

**Authors:** Chenxi Lin, Jia Li, Ye Deng, Xiongwen Li, Shirong Li

**Affiliations:** Department of Otorhinolaryngology, Meizhou People's Hospital, Meizhou City, Guangdong Province, China

**Keywords:** Adipocyte fatty acid-binding protein, Allergic rhinitis, Interleukin-6, Mendelian randomization, Obesity

## Abstract

**Objectives:**

Observational studies suggested that obesity may promote the development of allergic rhinitis. The aim of this study was to explore the association of obesity, lipids and adipokines with this allergic disease at the genetic level using Mendelian randomization strategies.

**Methods:**

Summary data for three obesity indicators (such as body mass index), eight lipid indicators (such as triglycerides) and six adipokines (such as interleukin-6 and adipocyte fatty acid-binding protein) were collected, and suitable instrumental variables were extracted from these summary data according to the three main assumptions of Mendelian randomization. Three Mendelian randomization methods (such as inverse variance weighted) were used to detect the casual effect of the above indicators on allergic rhinitis risk. Sensitivity analyses were performed to assess heterogeneity and horizontal pleiotropy.

**Results:**

After Bonferroni correction, the inverse variance weighted reported that elevated levels of interleukin-6 and adipocyte fatty acid-binding protein were nominally associated with the decreased risk of allergic rhinitis (OR = 0.870, 95% CI 0.765–0.990, *p* = 0.035; OR = 0.732, 95% CI 0.551–0.973, *p* = 0.032). The other Mendelian randomization methods supported these results. Obesity, lipids and other adipokines were not related to this allergic disease. Sensitivity analyses found no heterogeneity and horizontal pleiotropy in the study.

**Conclusion:**

The study provided some interesting, but not sufficient, evidence to suggest that interleukin-6 and adipocyte fatty acid-binding protein might play a protective role in the development of allergic rhinitis at the genetic level. These findings should be validated by more research.

**Level of evidence:**

This was a Mendelian randomized study with a level of evidence second only to clinical randomized trials, and higher than cohort and case-control studies.

## Highlights

• Obesity and lipids cannot genetically increase the risk of allergic rhinitis.
• Interleukin-6 might contribute to the development of this disease.
• Adipocyte fatty acid-binding protein perhaps promoted the onset of this disease.
• Bonferroni correction had the ability to eliminate these two correlations.
• The above findings can be instructive for future research on allergic rhinitis.

## Introduction

Allergic rhinitis is a common allergic and inflammatory disease in the upper respiratory tract.^1^ More than 500 million people are affected by this disease worldwide, and the prevalence’s of allergic rhinitis in China and several western countries are even higher, reaching between 12 and 30%.^2^, ^3^ Although the disease is not fatal, it can still cause a range of adverse effects if not effectively controlled. For example, severe allergic rhinitis may disrupt sleep, cause headaches and memory loss, trigger sinusitis and aggravate asthma.^4^, ^5^ Symptoms such as the runny nose induced by the disease can also affect the patient's image when socializing, causing social fear and depression.^6^ However, there is no cure for allergic rhinitis, and the available medications can only relieve the symptoms.^7^ Therefore, in-depth research into the pathogenesis and risk factors of the disease is necessary.

It is well known that obesity is a growing global health challenge. Several studies have confirmed the correlation between obesity and the development of asthma.^8^, ^9^ Because allergic rhinitis and asthma are very similar in their pathogenesis and there is a concept of “one airway, one disease”, the effect of obesity on allergic rhinitis has therefore attracted a great deal of attention globally.^10^ Then, an increasing number of studies have been carried out, suggesting that obesity and overweight may increase the risk of developing allergic rhinitis, and the correlation is implicated in inflammation and air pollution.^11^, ^12^, ^13^ However, these results are all obtained by observational studies and are susceptible to confounding factors and causal inversions. Therefore, a final conclusion still cannot be drawn.

Mendelian randomization (MR) studies are a form of genetics-based epidemiological research that allows for causal inference at the genetic level by replacing the traits under study with a series of genetic variants.^14^, ^15^ Such studies can effectively make up for the deficiencies of observational studies. However, there are still no published MR studies exploring the association between obesity and allergic rhinitis at present.

Taken together, this study proposed to adopt the MR approach to explore the impact of obesity on the risk of developing allergic rhinitis at the genetic level. Considering the close association of numerous peripheral lipids, lipoproteins, apolipoproteins and adipokines with obesity, this study also used this approach to explore the association of a range of lipids and adipokines with this allergic disease at the genetic level. The findings obtained from this study were expected to increase our knowledge and understanding of the pathogenesis of allergic rhinitis.

## Methods

### Summary data for exposures and outcome

Several obesity indicators, lipid indicators and adipokines were identified as the exposures in the study. The obesity indicators were Body Mass Index (BMI), Body Fat Percentage (BFP) and Waist-to-Hip Ratio (WHR). The lipid indicators were Triglycerides (TG), Total Cholesterol (TC), Low Density Lipoprotein (LDL) cholesterol, High Density Lipoprotein (HDL) cholesterol, non-HDL cholesterol, Lipoprotein A [Lp(a)], Apolipoprotein A-I (Apo A-I) and Apolipoprotein B (ApoB). The adipokines were Interleukin-6 (IL-6), Interleukin-8 (IL-8), Agouti-Related Protein (AGRP), Adipocyte Fatty Acid-binding Protein (A-FABP), leptin and resistin. In addition, allergic rhinitis was defined as the outcome of this study.

The summary data for BMI was obtained from a meta-analysis of Genome-Wide Association Studies (GWASs) for height and BMI in more than 700,000 individuals of European ancestry.^16^ The summary data for BFP was collected from a GWAS of the MRC Integrative Epidemiology Unit at the university of bristol (IEU) involving more than 450,000 individuals of European ancestry. The summary data for WHR was collected from a GWAS of the within family consortium including 85,978 individuals of European ancestry. The above summary data for BFP and WHR were downloaded from the IEU open GWAS project (https://gwas.mrcieu.ac.uk/), and the GWAS IDs were ukb-b-8909 and ieu-b-4830.

The summary data for TG, TC, LDL cholesterol, HDL cholesterol, non-HDL cholesterol were obtained from a series of lipid GWASs involving more than 1,300,000 individuals of European ancestry.^17^ The summary data for Lp(a) was collected from a GWAS of Neale lab using 273,896 individuals of European ancestry, and was also downloaded from the IEU open GWAS project with its GWAS ID of ukb-d-30790_raw. The summary data for Apo A-I and ApoB were obtained from a multivariable MR analysis evaluating the relationship between circulating apolipoproteins with risk of coronary heart disease using 393,193–439,214 individuals of European ancestry.^18^ The summary data for IL-6, IL-8, AGRP, A-FABP, leptin and resistin were collected from a GWAS for genomic and drug target evaluation of 90 cardiovascular proteins in 30,931 European individuals.^19^

The summary data for allergic rhinitis was obtained from a FinnGen study including 340,880 Finnish individuals.^20^ These subjects were identified by reviewing the ICD-10 codes in the hospitalization records, and the ICD-10 codes for allergic rhinitis were J30.10, J30.19, J30.2, J30.3 and J30.4.

The detailed characteristics of these traits were listed in Table 1.

**Table 1:** Characteristics of the summary data in the study.

Traits (outcome/exposures)	Sources	Years	Population	Sex	Sample size	SNPs^a^	F statistic
Outcome							
Allergic rhinitis	FinnGen	2022	European	Male and female	340,880	‒	‒
Obesity indicators							
Body mass index	PMID: 30124842	2018	European	Male and female	681,275	464	31.076
Body fat percentage	MRC-IEU	2018	European	Male and female	454,633	337	25.671
Waist-hip ratio	Within family consortium	2022	European	Male and female	85,978	16	17.360
Lipid indicators							
Triglycerides	PMID: 34887591	2021	European	Male and female	1,320,016	363	55.714
Total cholesterol	PMID: 34887591	2021	European	Male and female	1,320,016	333	77.232
LDL cholesterol	PMID: 34887591	2021	European	Male and female	1,320,016	297	66.435
HDL cholesterol	PMID: 34887591	2021	European	Male and female	1,320,016	412	60.039
non-HDL cholesterol	PMID: 34887591	2021	European	Male and female	1,320,016	274	62.811
Lipoprotein A	Neale lab	2018	European	Male and female	273,896	16	2350.190
Apolipoprotein A-I	PMID: 32203549	2020	European	Male and female	393,193	251	57.110
Apolipoprotein B	PMID: 32203549	2020	European	Male and female	439,214	149	58.843
Adipokines							
Interleukin-6	PMID: 33067605	2020	European	Male and female	21,758	15	12.016
Interleukin-8	PMID: 33067605	2020	European	Male and female	21,758	5	10.001
Agouti-related protein	PMID: 33067605	2020	European	Male and female	21,758	9	10.360
Fatty acid-binding protein	PMID: 33067605	2020	European	Male and female	21,758	10	10.158
Leptin	PMID: 33067605	2020	European	Male and female	21,758	14	10.017
Resistin	PMID: 33067605	2020	European	Male and female	21,758	41	10.003

MRC-IEU, MRC Integrative Epidemiology Unit at the University of Bristol (IEU); LDL, Low-Density Lipoprotein; HDL, High-Density Lipoprotein.

a The number of instrumental variables that satisfied the three assumptions of Mendelian randomization.

### Instrumental variables

Single Nucleotide Polymorphism (SNP) is a DNA sequence polymorphism caused by a variation in a single nucleotide at the genomic level.^21^ It is the most common type of human heritable variation, accounting for over 90% of all polymorphisms. In the present study, suitable SNPs were used as instrumental variables, which was extracted from the above summary data according to the three main assumptions of MR.^22^

To meet the correlation assumption, all included SNPs must have a genome-wide correlation *p*-value of less than 5 × 10^−8^ (for obesity and lipid indicators) or 5 × 10^−6^ (for adipokines), and their F-statistics must be greater than 10. To satisfy the independence assumption, all SNPs with Linkage Disequilibrium (LD) were directly excluded using a clumping window of 10 MB and an r^2^ cutoff of 0.001. To satisfy the exclusivity assumption, all SNPs that were significantly associated with confounders or outcomes were directly excluded by phenoscanner (http://www.phenoscanner.medschl.cam.ac.uk/), and the potential confounders included nasal polyp, bronchial asthma, otitis media, sinusitis, allergic strep throat and epistaxis.^1^

### Mendelian randomization analyses

This was a two-sample MR that adopted three main methods for causal inference, namely random-effect Inverse Variance Weighted (IVW), weighted median and MR-Egger.^23^, ^24^ Briefly, the IVW is by far the most accurate method for causal inference, when all SNPs are unaffected by horizontal pleiotropy. The other two methods are not as accurate but have wider applicability conditions. Of these, weighted median accepts that about 50% of SNPs are horizontally pleiotropic, while MR-Egger can provide results when all SNPs are affected by horizontal pleiotropy. The results of these two methods can be used as a complement to the IVW results. In the present study, all three methods reported Odds Ratios (ORs), 95% Confidence Intervals (95% CIs) and *p*-values. And correlations can be considered nominally significant when the *p* values for the IVW were less than 0.05 and the direction of the results obtained by the other two methods were the same as the direction of the IVW results. Due to the multiple comparison design (17 exposures and 1 outcome), Bonferroni-corrected *p*-values less than 0.003 (0.05/17) indicated statistical significance. In addition, scatter plots and forest plots were made to visualize the above results.

A series of sensitivity analyses were also performed.^25^ Heterogeneity among SNPs was assessed using Cochran’s Q test. Horizontal pleiotropy among SNPs was measured by MR-Egger intercept test and MR-PRESSO test. All above sensitivity analyses reported *p*-values, and the *p*-values less than 0.05 indicated heterogeneity or horizontal pleiotropy. Horizontal pleiotropy was also determined by funnel plot and leave-one-out test. If the funnel plot was relatively symmetrical, it suggested that there was no significant horizontal pleiotropy. And the leave-one-out test can eliminate each SNP in turn and observe the effect of each SNP on the pooled results.

All MR analyses were done using TwoSampleMR in R software (version 4.2.3).

## Results

### Sources of summary data in the study

In Table 1, there were seventeen exposures and one outcome in the study. Their summary data were all from European populations, containing subjects from both genders. The sample sizes were between 21,758 and 1,320,016. And a total of 5–464 suitable SNPs were selected for this study from the summary data of the seventeen exposures according to the three assumptions of MR described above, and they had F-statistics between 10.001 and 2350.190.

### Effect of obesity and lipids on the risk of allergic rhinitis

In Table 2, the IVW reported that BMI, BFP and WHR were not associated with the risk of allergic rhinitis (OR = 1.042, 95% CI 0.935–1.162, *p* = 0.452; OR = 1.053, 95% CI 0.901–1.230, *p* = 0.517; OR = 0.061, 95% CI 0.001–3.781, *p* = 0.184). The weighted median and MR-Egger supported these results obtained from the IVW (*p* > 0.05).

**Table 2:** Mendelian randomization estimates for obesity affecting on the risk of allergic rhinitis.

Exposures	MR methods	Nº of SNPs^a^	*p* for MR	OR (95% CI)	*p* for Cochran’s Q	*p* for MR-Egger intercept	*p* for MR-PRESSO
Body Mass Index							
	IVW	412	0.452	1.042 (0.935–1.162)	0.082	0.937	0.086
	Weighted median	412	0.660	1.036 (0.885–1.213)			
	MR-Egger	412	0.874	1.029 (0.726–1.457)			
Body fat percentage							
	IVW	286	0.517	1.053 (0.901–1.230)	0.069	0.755	0.073
	Weighted median	286	0.628	1.056 (0.848–1.314)			
	MR-Egger	286	0.635	1.150 (0.646–2.046)			
Waist-hip ratio							
	IVW	14	0.184	0.061 (0.001–3.781)	0.273	0.640	0.297
	Weighted median	14	0.585	0.216 (0.001–53.070)			
	MR-Egger	14	0.856	0.426 (0.001–3605.636)			

SNP, Single Nucleotide Polymorphisms; MR, Mendelian randomization; OR, Odds Ratio; CI, Confidence Interval; IVW, Inverse-Variance Weighted.

a Number of SNPs used for causal inference in Mendelian randomization.

In Table 3, the IVW also reported that TG, TC, LDL cholesterol, HDL cholesterol, non-HDL cholesterol, Lp(a), Apo A-I and ApoB were not related to the risk of the disease (OR = 1.045, 95% CI 0.935–1.167, *p* = 0.436; OR = 1.002, 95% CI = 0.906–1.107, *p* = 0.976; OR = 0.961, 95% CI 0.869–1.063, *p* = 0.441; OR = 0.955, 95% CI 0.873–1.046, *p* = 0.322; OR = 0.980, 95% CI 0.886–1.084, *p* = 0.696; OR = 1.000, 95% CI 0.999–1.001, *p* = 0.587; OR = 0.923, 95% CI 0.831–1.025, *p* = 0.136; OR = 1.016, 95% CI 0.922–1.120, *p* = 0.751). And the weighted median and MR-Egger also provided some similar results (*p* > 0.05).

**Table 3:** Mendelian randomization estimates for blood lipids affecting on the risk of allergic rhinitis.

Exposures	MR methods	Nº of SNPs^a^	*p* for MR	OR (95% CI)	*p* for Cochran’s Q	*p* for MR-Egger intercept	*p* for MR-PRESSO
Triglycerides							
	IVW	306	0.436	1.045 (0.935–1.167)	0.084	0.568	0.093
	Weighted median	306	0.773	1.027 (0.855–1.235)			
	MR-Egger	306	0.979	0.997 (0.822–1.211)			
Total cholesterol							
	IVW	277	0.976	1.002 (0.906–1.107)	0.081	0.109	0.090
	Weighted median	277	0.846	0.985 (0.847–1.145)			
	MR-Egger	277	0.206	0.898 (0.760–1.061)			
LDL cholesterol							
	IVW	253	0.441	0.961 (0.869–1.063)	0.088	0.539	0.097
	Weighted median	253	0.327	0.926 (0.795–1.079)			
	MR-Egger	253	0.336	0.925 (0.790–1.084)			
HDL cholesterol							
	IVW	355	0.322	0.955 (0.873–1.046)	0.061	0.137	0.061
	Weighted median	355	0.249	0.922 (0.803–1.059)			
	MR-Egger	355	0.077	0.883 (0.769–1.013)			
non-HDL cholesterol							
	IVW	228	0.696	0.980 (0.886–1.084)	0.073	0.956	0.085
	Weighted median	228	0.654	0.968 (0.840–1.115)			
	MR-Egger	228	0.851	0.984 (0.831–1.165)			
Lipoprotein A							
	IVW	13	0.587	1.000 (0.999–1.001)	0.914	0.892	0.947
	Weighted median	13	0.554	1.000 (0.999–1.001)			
	MR-Egger	13	0.617	1.000 (0.999–1.001)			
Apolipoprotein A-I							
	IVW	189	0.136	0.923 (0.831–1.025)	0.156	0.652	0.163
	Weighted median	189	0.452	0.940 (0.799–1.105)			
	MR-Egger	189	0.233	0.890 (0.735–1.077)			
Apolipoprotein B							
	IVW	123	0.751	1.016 (0.922–1.120)	0.071	0.928	0.079
	Weighted median	123	0.607	1.037 (0.904–1.188)			
	MR-Egger	123	0.882	1.011 (0.876–1.167)			

SNP, Single Nucleotide Polymorphisms; MR, Mendelian randomization; OR, Odds Ratio; CI, Confidence Interval; IVW, Inverse-Variance Weighted; LDL, Low-Density Lipoprotein; HDL, High-Density Lipoprotein.

a Number of SNPs used for causal inference in Mendelian randomization.

### Effect of adipokines on the risk of allergic rhinitis

**Figure 1:**
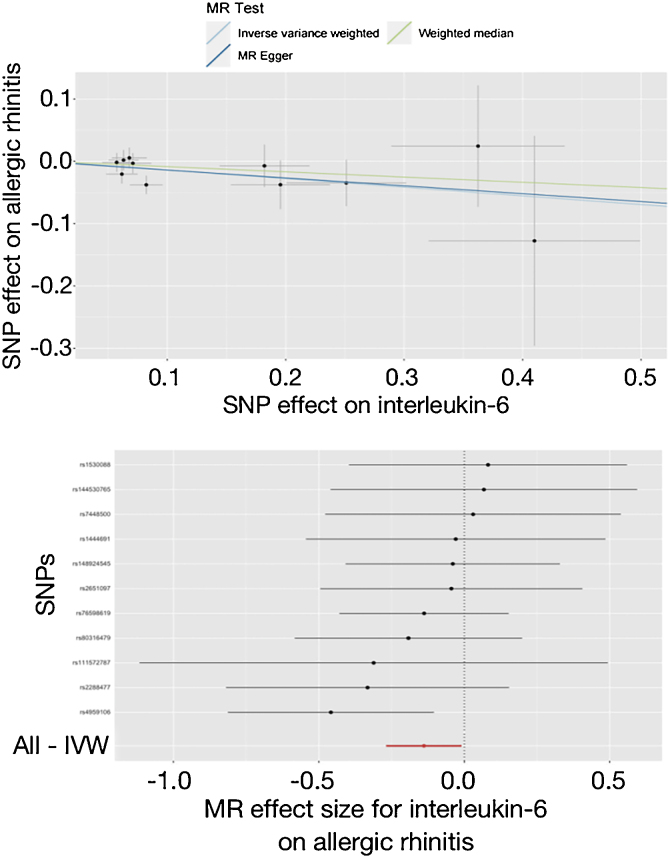
Effect of interleukin-6 on allergic rhinitis in the study. SNP, Single Nucleotide Polymorphisms; MR, Mendelian randomization; IVW, Inverse-Variance Weighted.

In Table 4, the IVW reported that elevated level of IL-6 was nominally associated with the decreased risk of allergic rhinitis (OR = 0.870, 95% CI 0.765–0.990, *p* = 0.035). Though the results of the weighted median and MR-Egger were not statistically significant (OR = 0.919, 95% CI 0.770–1.097, *p* = 0.351; OR = 0.881, 95% CI 0.679–1.143, *p* = 0.365), they were in the same direction as the IVW results. In Fig. 1, the scatter and forest plots visualized the above results. In Table 4, the Cochran’s Q did not find any heterogeneity (*p* = 0.777), and the MR-Egger intercept and MR-PRESSO did not report any horizontal pleiotropy or outliers (*p* = 0.918, *p* = 0.784). In Supplemental Fig. 12, the leave-one-out test also did not detect any significant outliers.

**Table 4:** Mendelian randomization estimates for adipokines affecting on the risk of allergic rhinitis.

Exposures	MR methods	Nº of SNPs^a^	*p* for MR	OR (95% CI)	*p* for Cochran’s Q	*p* for MR-Egger intercept	*p* for MR-PRESSO
Interleukin-6							
	IVW	11	0.035	0.870 (0.765–0.990)	0.777	0.918	0.784
	Weighted median	11	0.351	0.919 (0.770–1.097)			
	MR-Egger	11	0.365	0.881 (0.679–1.143)			
Interleukin-8							
	IVW	4	0.280	1.146 (0.895–1.467)	0.954	0.931	0.951
	Weighted median	4	0.416	1.124 (0.848–1.489)			
	MR-Egger	4	0.928	1.073 (0.278–4.144)			
Agouti-related protein							
	IVW	7	0.172	0.842 (0.658–1.078)	0.183	0.597	0.211
	Weighted median	7	0.646	0.935 (0.704–1.244)			
	MR-Egger	7	0.657	2.442 (0.060–100.176)			
Fatty acid-binding protein							
	IVW	4	0.032	0.732 (0.551–0.973)	0.398	0.581	0.466
	Weighted median	4	0.093	0.737 (0.517–1.052)			
	MR-Egger	4	0.427	0.411 (0.070–2.402)			
Leptin							
	IVW	34	0.773	1.014 (0.923–1.113)	0.150	0.857	0.143
	Weighted median	34	0.472	1.049 (0.921–1.194)			
	MR-Egger	34	0.987	0.998 (0.824–1.209)			
Resistin							
	IVW	12	0.283	1.122 (0.909–1.384)	0.064	0.353	0.068
	Weighted median	12	0.792	1.032 (0.814–1.309)			
	MR-Egger	12	0.295	2.495 (0.493–12.625)			

SNP, Single Nucleotide Polymorphisms; MR, Mendelian randomization; OR, Odds Ratio; CI, Confidence Interval; IVW, Inverse-Variance Weighted.

a Number of SNPs used for causal inference in Mendelian randomization.

**Figure 2:**
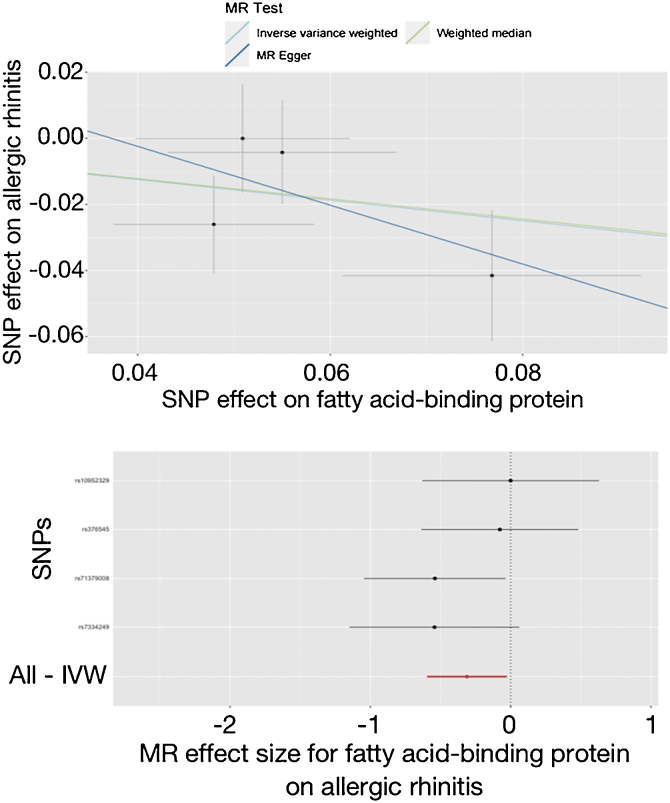
Effect of adipocyte fatty acid-binding protein on allergic rhinitis in the study. SNP, Single Nucleotide Polymorphisms; MR, Mendelian randomization; IVW, Inverse-Variance Weighted.

In Table 4, the IVW reported that higher level of A-FABP was nominally associated with the lower risk of allergic rhinitis (OR = 0.732, 95% CI 0.551–0.973, *p* = 0.032). The weighted median and MR-Egger results were in the same direction as the IVW direction (OR = 0.737, 95% CI 0.517–1.052, *p* = 0.093; OR = 0.411, 95% CI 0.070–2.402, *p* = 0.427). These results were visualized in Fig. 2. The Cochran’s Q, MR-Egger intercept and MR-PRESSO did not report any heterogeneity or horizontal pleiotropy in Table 4 (*p* = 0.398, *p* = 0.581, *p* = 0.466). The leave-one-out test did not find any outliers in Supplemental Fig. 15.

In addition, the IVW did not detect any association of the other adipokines with the risk of allergic rhinitis (*p* > 0.05).

## Discussion

As an allergic and inflammatory disease, the etiology and pathogenesis of allergic rhinitis are still unknown. However, genetic background is supposed to be one of the main pathogenic mechanisms. Thus, we investigated the genetic effects of obesity, lipids and adipokines on the risk of the disease using MR methods in the study, which contributed to our further understanding of the risk factors and pathogenesis of allergic rhinitis, and helped us to find suitable therapeutic targets and improve therapeutic strategies so as to reduce the incidence of this disease and improve its prognosis.

According to the IVW results in this study, although all obesity and lipid indicators cannot influence the risk of developing allergic rhinitis, the levels of two adipokines were potentially correlated with the risk of the disease. Briefly, for each SD increase in peripheral levels of IL-6 and A-FABP, the risk of developing allergic rhinitis decreased by approximately 10%–30%. Because the sensitivity analyses did not detect horizontal pleiotropy, the IVW results mentioned above were considered to be the most reliable. Moreover, the results obtained by the other two methods were directionally consistent with the IVW results, although none of them were statistically significant. Thus, MR can preliminarily confirmed the effect of IL-6 and A-FABP on the risk of allergic rhinitis. However, it should be noted that the IVW *p*-values for these two adipokines were only slightly lower than 0.05. More importantly, there was a design of multiple comparisons in this study. Therefore, we believed that these analyses had the potential for type I error, and therefore the Bonferroni correction was adopted. After the correction, the results showed that these two adipokines were only nominally associated with the risk of allergic rhinitis. These results were not sufficient to draw a conclusion, but they were certainly helpful for future research.

As mentioned above, several observational studies had reported that obesity contributed to the development of allergic rhinitis.^11^, ^12^, ^13^ But, the results of the present study at the genetic level did not support these findings. Considering that the pathogenesis of this allergic disease was complex, it certainly included multiple genetic and environmental factors. So, it was still possible that obesity may have an impact on the risk of developing allergic rhinitis through phenotypic-level mechanisms.

IL-6 was a classical inflammatory factor that can be secreted by several types of cells. In the peripheral circulation, approximately 1/3 of this factor was derived from adipose tissue and therefore IL-6 was also considered as an adipokine with inflammatory regulatory functions. Two previous observational studies found that there were rs1800795 (G/C at −174) and rs1800796 (G/A at −597) polymorphisms in IL-6 and that rs1800795 was associated with allergic rhinitis risk.^26^, ^27^ Of them, the study from China reported that individuals expressing the C allele of rs1800795 were at higher risk of developing this allergic disease, whereas the other study from Middle Asia concluded that individuals expressing the G allele were more susceptible to allergic rhinitis.^26^, ^27^ These contradictory results may be explained by the different study populations. Meanwhile, the present study included a European population and reported that peripheral IL-6 levels may nominally reduce the risk of this allergic disease, which did not consider genetic polymorphisms due to insufficient data. So, both the present MR study and the two observational studies mentioned above suggested that IL-6 had the potential to affect the development of allergic rhinitis, and the mechanism might be influenced by race and genetic polymorphism.

FABP were fatty acid carriers in a wide range of cells (including adipocytes) and played an important role in the physiological use of fatty acids in these cells. A previous animal study using mice explained the role of this proteins in allergic lung inflammation.^28^ Briefly, knockdown of FABP in the lungs can cause severe allergic lung inflammation, and up-regulation of this proteins suppressed allergy-associated immune cell activity. In addition, a high-fat diet may result in down-regulation of FABP expression in the lungs. The present study investigated the correlation between peripheral A-FABP levels and allergic rhinitis, and the obtained results appeared to be partially consistent with this animal study.

The main advantage of this study is the MR design. Compared to observational studies, this study allowed causal inferences to be made at the genetic level between the exposures and the outcome, avoiding many confounding factors and the appearance of causal inversions. So, the results obtained from this study were more reliable. Meanwhile, there were still some limitations in this study. For example, IL-6 was polymorphic, and it was likely that this polymorphism had a significant impact on allergic rhinitis. Also, closely related to this issue was the speculation that the effect of IL-6 on allergic rhinitis may be ethnospecific. In addition, the number of instrumental variables for several exposures (i.e., adipokines) might be somewhat inadequate due to the lack of sufficient summary data. All of these questions remained to be explored in future studies.

## Conclusion

The present study provided some interesting, but not sufficient, evidence to suggest that IL-6 and A-FABP might play a protective role in the development of allergic rhinitis at the genetic level. However, it was important to emphasize that this was not a final conclusion, and the above findings should be validated by more research. In addition, the study did not find a genetic association of obesity or peripheral lipid levels with this allergic disease. These studies helped us to objectively validate previous observational studies and deepen our understanding of the risk factors and pathogenesis of allergic rhinitis.

## Funding

Not applicable.

## Conflicts of interest

The authors declare no conflicts of interest.
