# Supplementary material for: Effect of obesity, lipids and adipokines on allergic rhinitis risk: a Mendelian randomization study
Source: Braz J Otorhinolaryngol. 2023 Aug 17;89(5):101306. doi: 10.1016/j.bjorl.2023.101306 (PMC10472243; doi:10.1016/j.bjorl.2023.101306)
Supplement: Supplementary file 1 [file mmc1.docx]

**BJORL-D-23-00108 -** **Supplementary Material**

**Supplemental Figure 1** Effect of body mass index on the risk of allergic rhinitis.

**Supplemental Figure 2** Effect of body fat percentage on the risk of allergic rhinitis.

**Supplemental Figure 3** Effect of waist-hip ratio on the risk of allergic rhinitis.

**Supplemental Figure 4** Effect of triglycerides on the risk of allergic rhinitis.

**Supplemental Figure 5** Effect of total cholesterol on the risk of allergic rhinitis.

**Supplemental Figure 6** Effect of LDL cholesterol on the risk of allergic rhinitis.

**Supplemental Figure 7** Effect of HDL cholesterol on the risk of allergic rhinitis.

**Supplemental Figure 8** Effect of non-HDL cholesterol on the risk of allergic rhinitis.

**Supplemental Figure 9** Effect of lipoprotein A on the risk of allergic rhinitis.

**Supplemental Figure 10** Effect of apolipoprotein A-I on the risk of allergic rhinitis.

**Supplemental Figure 11** Effect of apolipoprotein B on the risk of allergic rhinitis.

**Supplemental Figure 12** Effect of interleukin-6 on the risk of allergic rhinitis.

**Supplemental Figure 13** Effect of interleukin-8 on the risk of allergic rhinitis.

**Supplemental Figure 14** Effect of agouti-related protein on the risk of allergic rhinitis.

**Supplemental Figure 15** Effect of adipocyte fatty acid-binding protein on the risk of allergic rhinitis.

**Supplemental Figure 16** Effect of leptin on the risk of allergic rhinitis.

**Supplemental Figure 17** Effect of resistin on the risk of allergic rhinitis.
